# Henrietta Lacks and America’s dark history of research involving African Americans

**DOI:** 10.1002/nop2.1257

**Published:** 2022-06-14

**Authors:** Diana‐Lyn Baptiste, Nicole Caviness‐Ashe, Nia Josiah, Yvonne Commodore‐Mensah, Joyell Arscott, Patty R. Wilson, Shaquita Starks

**Affiliations:** ^1^ Johns Hopkins School of Nursing Baltimore Maryland USA; ^2^ Duke University School of Nursing Durham North Carolina USA; ^3^ Columbia University School of Nursing New York New York USA; ^4^ SAMHSA Minority Fellowship Program Rockville Maryland USA; ^5^ Department of Epidemiology Johns Hopkins Bloomberg School of Public Health Baltimore Maryland USA; ^6^ Department of HIV Epidemiology and Prevention Science Johns Hopkins Bloomberg School of Public Health Baltimore Maryland USA; ^7^ Emory Nell Hodgson Woodruff School of Nursing Atlanta Georgia USA

HeLa cells came from an African American woman who was flesh and blood, who had a family and who had a story.‐ Jeri Lacks‐Whye (granddaughter of Henrietta Lacks).

## 1 A DARK HISTORY OF RESEARCH

The daunting truth throughout America’s dark history and present practice of American research is deeply rooted in anti‐Black racism. Research in the U.S. has been used throughout history to justify and rationalize the enslavement and experimentation of African Americans (Black people), and this racist legacy abides through race‐based medicine (Lujan & DiCarlo, 2018). Race‐based medicine has been used to facilitate incidents of racial injustice, unethical research and practices infringed on African American populations spanning over 400 years (Cerdeña et al., 2020). We echo the assertion of Cerdeña et al. (2020) that “…race is a poor proxy for human variation. Physical characteristics used to identify racial groups vary with geography and do not correspond to underlying biological traits” (Cerdeña et al., 2020). Thus, race is not a biological reality but a social construct. Race as biology is fiction; racism as a social problem is real (Lujan & DiCarlo, 2018).

This editorial aims to increase awareness to ongoing quandary over unethical scientific research practices that have, and continue to dehumanize and devalue Black lives. At the forefront of this discussion highlights Henrietta Lacks' cancer cells, which were taken without informed consent and still used today for medical research. We boldly say her name and unapologetically share the lesser‐known accounts of Black people subjected to unethical human experimentation throughout American history.

Among nurses, knowledge and awareness of the history of unethical research is nothing new. For several decades we have learned about the Tuskegee Study of Untreated Syphilis in the Negro Male (TSUS), which involved 600 African American men with syphilis who were told they had “bad blood” (Gamble, 1997; Tuskegee University, 2022). The goal of this study was to observe the progression of the disease, when there was no known effective treatment. During the study, penicillin was later identified as an appropriate treatment for syphilis; however, treatment was withheld while researchers continued observing the progression of the disease among participants. More than 200 of the study participants who did not receive treatment suffered from serious complications such as blindness, insanity, and death (Tuskegee University, 2022). Similar to the TSUS, countless other unethical practices have impacted marginalized persons, specifically those who are African American (Gamble, 1997). Lesser‐known unethical research activities involved African Americans as early as the 1800's and as recently as the 1990's. Unfortunately, there is relatively little discussion surrounding this very dark era in research and its current implications, beyond the purposes of learning research ethics.

The first known examples of unethical research practices were documented around the 1840's, when several young enslaved African American women received experimental gynaecological procedures and surgeries without anaesthesia from gynaecologist, Dr. James Marion Sims (Gamble, 1997; Sims, 1884). Sims repeated these painful procedures multiple times on three documented enslaved African American teenage girls, of whom he purchased, who are only known as Anarcha, Betsy and Lucy (Sims, 1884). Sim’s autobiography recounts the gynaecological procedure, vesicovaginal fistula repair, that Anarcha received more than 30 times, where Betsy reportedly was restrained and forced to endure prolonged tortuous operations as well. Sims documented Lucy’s agony as “extreme,” since her suffering seemed to be near death, while he deemed her as “heroic and brave” (Sims, 1884). Sims invented the vaginal speculum from these unconsented experiments which shielded white women from painful experimental procedures (Gamble, 1997). Sims was highly revered for his work in changing gynaecology practice, but his techniques that were used to develop these innovative methods have had little attention or critique. [Correction added on 23 July 2022, after first online publication: In the preceding sentence, the spelling of the name “Marion” was corrected.]

A more recent example of unethical research practices involving African Americans occurred in the late 1990s, when thirty‐four African American and Hispanic boys from ages 6–10 living in New York City were recruited in a study to determine if ingestion of the drug Fen‐Phen™ (fenfluramine) was associated with aggressive behaviours (Brown, 2003). The boys included in this study were from impoverished families, with no pre‐existing behavioural problems (Pine et al., 1997). Although the boys' parents consented, they were not given informed consent; thus, were not fully aware of the risk and consequences of the experiment (Brown, 2003). Throughout the study, the boys were placed on a low‐protein diet, not allowed water intake during overnight sessions and were not given any prescribed maintenance medications for underlying chronic conditions, such as asthma (Brown, 2003). Even after the drug was deemed dangerous due to causing severe heart valve problems and banned in 1997 for use in adult patients, this experiment continued for another year (Brown, 2003). The actions taken to conduct this research on these young boys were abysmal, and the long‐term impact remains unclear. The research was done solely to test a hypothesis with no therapeutic benefit to these boys, their families, nor their communities. When researchers work with marginalized groups without leaving sustainable solutions, feelings of being used, and abandonment can ensue, which can breed iatrophobia among these vulnerable individuals (Gamble, 1997; Skloot, 2010; Sodeke & Powell, 2019). The reckoning of the above incidents challenges history’s invisibility of these unethical research practices. We now focus our attention on Henrietta Lacks, whose cells serve as an example of unethical experimentation, and still have a lasting impact today.

## 2 HENRIETTA LACKS

“HeLa” cells, named after **He**nrietta **La**cks, are well known among those who study in the biological sciences, but very few know the story of the woman and her family who are attached to these cells. Like the authors of this editorial, many nurses were first introduced to Henrietta Lacks' story through Rebecca Skloot’s New York Times’ bestselling book, *“The Immortal Life of Henrietta Lacks,”* published in 2010. [Correction added on 23 July 2022, after first online publication: In the preceding sentence, the missing words “of this” were added.] Skloot’s book provides a very personal account of the experiences of Henrietta as she sought care at the Johns Hopkins Hospital in the 1950's for what would later be discovered as cervical cancer (Skloot, 2010). This story focuses not only on the flawed practices of cell retrieval, biobanking, and lack of informed consent, but also the lasting impact these practices had on one poor Black family in Baltimore, Maryland. This story shook an entire community contributing to ongoing, and lasting historical trauma for African Americans who currently live in Maryland.

Henrietta Lacks was an African American woman, born 1 August 1920 and died at the age of 31 from cervical cancer at the Johns Hopkins Hospital in Baltimore, Maryland. Henrietta came from a poor family of tobacco farmers in rural Virginia. She moved to the more industrialized city of Baltimore with her husband to raise five young children in 1941 (Skloot, 2010). When Henrietta arrived at Johns Hopkins Hospital for treatment of “ceaseless vaginal bleeding,” she did not know her cervical cells would be used for future scientific experiments since there was no informed consent detailing this use (Skloot, 2010; Sodeke & Powell, 2019). Researchers have since had access to HeLa cells and continue to use them, many unaware of the history or the woman behind the cells (Skloot, 2010).

HeLa cells were the first documented human cell line that divided easily and indefinitely, making monumental contributions to science, medicine, and nursing (Skloot, 2010). In 1953, the growth pattern and immortality of the cell line proved advantageous in understanding how the poliovirus replicates, infects, and causes poliomyelitis (Johns Hopkins Medicine, 2022; Skloot, 2010; Sodeke & Powell, 2019). Two years later, HeLa cells were used to study the impact of radiation on human cells and develop a new research method to create a more homogenous mutant colony by isolating a single mutant cell (Skloot, 2010). In 1956, the HeLa cells were used to document the behaviour of cancer cells helping researchers understand how malignant cells respond to pharmacological agents, viruses, and radiation (Johns Hopkins Medicine, 2022; Skloot, 2010; Sodeke & Powell, 2019). By 1960, HeLa cells were transported outer space to study the impact of radiation and space travel on the human body (Johns Hopkins Medicine, 2022; Skloot, 2010). The vast use of HeLa cells since Henrietta’s death is immeasurable and has impacted the progression of modern medicine on earth and beyond.

## 3 HELA CELLS TODAY

HeLa cell lines continue to thrive to date, helping with the advancement of science and medicine. The cell line has been used to make discoveries for cancer, HIV, Ebola and tuberculosis in‐vitro fertilization (Sodeke & Powell, 2019). More recently, HeLa cells have been used for human genome studies, virology and for the development of the COVID‐19 vaccine (Johns Hopkins Medicine, 2022). Henrietta’s family learned of the use of these cells about 20 years ago, even though the HeLa cell line has been used for over 60 years (Skloot, 2010). The family was traumatized by this revelation and have sought legal action; however, they are yet to receive compensation. This fact is appalling since pharmaceutical companies and other science organizations have profited billions from the use of Henrietta’s cells (Skloot, 2010). To rectify past injustices, the National Institutes of Health (NIH) established a HeLa Genome Data Use Agreement requiring researchers to deposit genome HeLa sequencing data in a controlled‐access database (Sodeke & Powell, 2019). Members of the Lacks’ family serve as members of the proposal review committee. Additionally, the NIH has recommended that the family be acknowledged in any publication that uses full genomic data from HeLa cells (Sodeke & Powell, 2019). This is a moral and ethical victory for the Lacks’ family.

A fitting tribute was made by her grandson, Alfred Lacks‐Carter, who recognized the important fact that his grandmother’s cells advanced cancer research—given that Lacks died of the disease. Lacks‐Carter states, *“They were taken in a bad way but they are doing good for the world,”* and *“they do so for people of all ethnicities”* (“Henrietta Lacks: science must right a historical wrong,”, 2020).

## 4 IMPLICATION FOR NURSES

Incidents such as those presented in this editorial have contributed to the lack of trust in research and medical mistrust among African Americans. The legacy of these trials continues to this day. Whether or not we have known about or were involved in these trials, we still feel the residual impact of these unethical practices. We understand existing barriers to develop rapport with patients and with recruiting participants for research, specifically those from racial and ethnic minority communities. These accounts are few among many examples of how Black people and their communities have been withheld from treatment and unjustly used for research. Each example presented shows the lack of ethical practice and a failure to recognize the autonomy and rights of Black people in research.

The authors of this editorial emphasize the need for heightened awareness of how unethical research practices affect marginalized populations. We call for nurses to incorporate cultural humility and compassion into clinical and research practice, and to develop an understanding of how historical trauma overshadows clinical practice, education, and research. Nurses cannot ignore the historical and ongoing impact of unethical research practices. Our ethical moral commitments as nurse researchers require us to have a sophisticated knowledge of patient’s rights and informed consent to enable us to treat all patients and participants equitably, and humanely, honouring bioethical sensitivity. Nurse researchers have the obligation to apply ethical and moral principles of beneficence, nonmaleficence, autonomy, and social justice for all persons. As we work to heal from the impact of a dark past in medical research, we challenge healthcare professionals to uphold these standard ethical principles.

Remembering the story of Henrietta Lacks, we honour those who, such as Henrietta, made major contributions to scientific knowledge without consent. In honouring her memory, we pledge to ensure that future generations of African Americans and ethnic minority people are not harmed, are informed and are protected from unethical practices.

## CONFLICTS OF INTEREST

The authors declare no conflict of interest.

## Data Availability

Data sharing is not applicable to this article as no new data were created or analyzed in this study.
